# Atrioesophageal Fistula: A Rare but Dangerous Complication From Catheter Ablation

**DOI:** 10.14309/crj.0000000000001209

**Published:** 2023-11-29

**Authors:** Scott Ventre, Peter Dellatore, Anish Vinit Patel

**Affiliations:** 1 Department of Medicine, Rutgers Robert Wood Johnson Medical School, New Brunswick, NJ; 2 Division of Gastroenterology & Hepatology, Rutgers Robert Wood Johnson Medical School, New Brunswick, NJ

**Keywords:** atrio-esophageal fistula, upper gastrointestinal bleeding, esophageal fistula

## Abstract

Atrioesophageal fistula is a rare complication of catheter ablation. It can be discovered on computerized topography of the chest. It is a difficulty entity to diagnose and treat and carries a mortality between 67% and 100%. Management options include surgical repair and esophageal stenting. We report here a rare case of an atrioesophageal fistula that presented with massive upper gastrointestinal bleeding and hemiparalysis.

## INTRODUCTION

Most cases that involve large-volume upper gastrointestinal bleeding are attributed to peptic ulcer disease, Mallory-Weiss tear, and esophageal varices. In rare occasions, fistulization to adjacent vascular strictures including major blood vessels such as the aorta can result in massive bleeding. Even rarer is the formation of an atrioesophageal fistula (AEF), which is a feared complication of catheter ablation with incidence <0.1%. The symptoms of AEF may occur between 1 and 4 weeks after procedure and may appear generally nonspecific, including fatigue, nausea, vomiting, chest discomfort, dysphagia, or odynophagia. A contrast-enhanced chest computed tomography (CT) is the best initial diagnostic tool to investigate for an AEF.

## CASE REPORT

**Figure 1.**
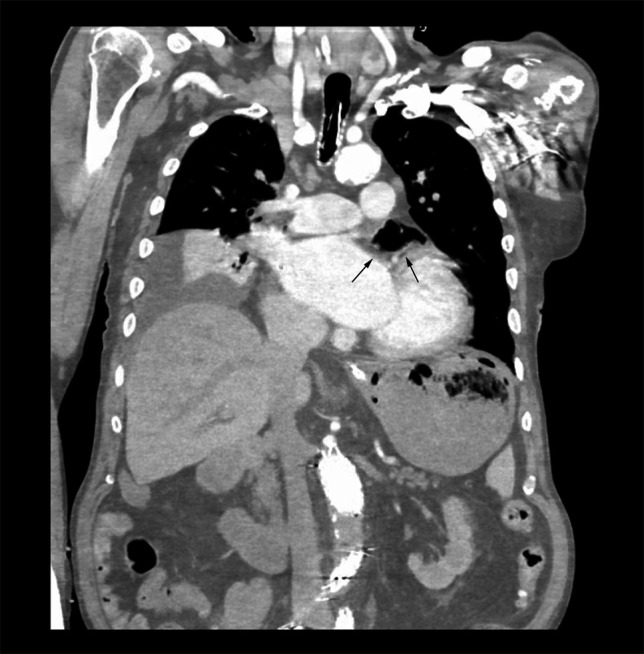
Computed tomography angiogram of the chest and abdomen demonstrated large amounts of air in the left atrium and small amounts in the left ventricle and right atrium.

**Figure 2.**
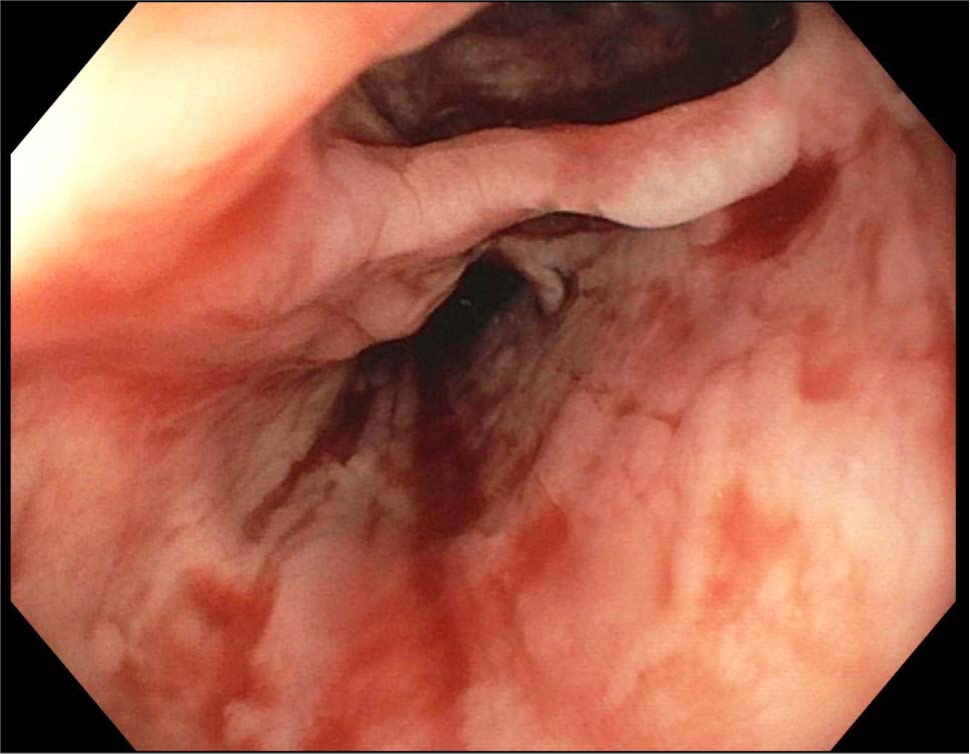
Esophagogastroduodenoscopy identified an 8-mm mucosal defect in the midesophagus with fistulization to an underlying pulsatile organ. This was consistent with an atrioesophageal fistula.

An 80-year-old White man with congestive heart failure and atrial fibrillation presented with large-volume hematemesis, acute right hemiparalysis, and hemorrhagic shock. He underwent catheter ablation performed 18 days earlier for treatment of electrical storm with ventricular tachycardia. On arrival, he was found to be minimally responsive requiring immediate endotracheal intubation and sedation. Initial hemoglobin was 8.3 g/dL. CT of the head did not reveal acute intracranial hemorrhage or pneumocephalus. CT angiogram of the chest and abdomen did not reveal active gastrointestinal bleeding. It demonstrated large amounts of air in the left atrium (LA) (Figure 1) and small amounts in the left ventricle and right atrium. However, because no obvious fistula was identified, these findings were attributed to central venous catheter placement in the emergency department. Although on the differential, because AEF could not be definitively established as the source of bleeding based on these findings, the patient was brought for esophagogastroduodenoscopy (EGD) within 6 hours of initial presentation. The EGD ultimately identified an 8-mm mucosal defect in the midesophagus with fistulization to an underlying pulsatile organ (Figure 2, Video 1). There was a large clot burden in the stomach that was evacuated, but no gastric pathology was identified. These findings, in conjunction with the recent catheter ablation, were consistent with an AEF. The patient was deemed to be of untenable risk of open surgical repair, and given the absence of other management alternatives and by family request, he was transitioned to comfort measures.

## DISCUSSION

Although they are rare, gastrointestinal complications may occur after catheter ablation. From mild esophageal irritation to severe gastric hypomotility because of vagal plexus damage, the degree and nature of injury can vary greatly.^1^ AEF, however, is among the most feared complications of this increasingly common procedure. With an incidence <0.1% of those undergoing catheter ablation, there is reason to believe that the incidence may be higher because of under-recognition and misdiagnosis.^2,3^ AEF formation is due in no small part to the close proximity of the LA to the esophagus. LA dilatation (commonly seen in patients undergoing ablation) imposes a broader contact area between the LA and esophagus and has been associated with a thinner fat pad.^1^ This may allow high levels of thermal energy to reach the esophagus and its surrounding structures. Excessive heat in the esophageal lumen causes ulceration and, in some cases, transmural ischemic necrosis because of damage to the esophageal microvasculature.^4^ This, in conjunction with other factors such as acid reflux and gastric hypomotility, may contribute to the progression to AEF.

The 3 most common symptoms of an AEF include fever, neurologic symptoms in the setting of cerebral air embolism, and hematemesis.^5^ Of note, a neurologic presentation such as stroke may be the initial or only manifestation of an AEF. A contrast-enhanced chest CT is the best initial diagnostic tool to evaluate for AEF. EGD should be avoided if at all possible when AEF is suspected because procedural air insufflation may introduce further air emboli into the vasculature. In the case presented above, the patient continued to worsen but had no identifiable active bleeding or fistulous connection on CT, even on further review. In these situations, EGD is often the only reasonable next step for diagnosing and treating the cause. Once AEF is identified, urgent surgical repair is considered the mainstay of treatment and offers the best chance for survival. In a systematic review by Han et al,^6^ esophageal stenting has shown its potential as a standalone treatment for patients unfit for surgical repair, with improved survival compared with those who did not undergo intervention. However, esophageal stenting remained inferior to surgical repair with regards to survival. Before this review, stenting has rarely been shown to be successful, unless used only as a bridge to surgical repair.^7^ Data regarding other more definitive endoscopic therapies for AEF are also limited. Two recent case reports describe successful endoscopic fistula closure through over-the-scope clip placement, one as a hybrid maneuver with preemptive cardiac perforation repair, and the other as an isolated definitive therapy.^8,9^ Despite these successes, the overall safety, durability, and predictors of success for this modality are not known and would require further study. In the case of our above patient, the surgical consultants agreed that given the nature of his AEF as well as his critical condition and comorbidities, endoscopic therapy was not a viable option.

Data continue to be limited as to how to prevent AEF formation. Although evidence is limited, it stands to reason that proton pump inhibitors (PPIs) administered before and after procedure may prevent progression of ulcerative lesions that occur after ablations. The patient in the case above developed an AEF, despite being placed on a PPI. Regardless, the theoretical benefits of PPIs likely outweigh the risk of adverse effects from short-term use and remain an attractive and worthwhile option.^10,11^ Ultimately, AEF is a difficult entity to prevent, diagnose, and treat. Despite its rarity, it carries a fatality rate between 67% and 100%. It is thus imperative to maintain a high index of suspicion for AEF under the right clinical circumstances.^12^

## DISCLOSURES

Author contributions: All authors contributed to the concept, design, drafting, revision, and final approval of the article. AV Patel is the article guarantor.

Financial disclosure: None to report.

Informed consent was obtained for this case report.
